# Stem cell modeling of mitochondrial parkinsonism reveals key functions of OPA1

**DOI:** 10.1002/ana.25221

**Published:** 2018-04-25

**Authors:** Mindaugas Jonikas, Martin Madill, Alexandre Mathy, Theresa Zekoll, Christos E. Zois, Simon Wigfield, Marzena Kurzawa‐Akanbi, Cathy Browne, David Sims, Patrick F. Chinnery, Sally A. Cowley, George K. Tofaris

**Affiliations:** ^1^ Nuffield Department of Clinical Neurosciences University of Oxford Oxford United Kingdom; ^2^ Weatherall Institute of Molecular Medicine, Department of Oncology University of Oxford Oxford United Kingdom; ^3^ Mitochondrial Research Group University of Newcastle Upon Tyne Newcastle Upon Tyne United Kingdom; ^4^ James Martin Stem Cell Facility, Sir William Dunn School of Pathology University of Oxford Oxford United Kingdom; ^5^ MRC Computational Genomics Analysis and Training Programme, MRC WIMM Centre for Computational Biology, MRC Weatherall Institute of Molecular Medicine University of Oxford Oxford United Kingdom; ^6^ Department of Clinical Neurosciences University of Cambridge Cambridge United Kingdom; ^7^ MRC Mitochondrial Biology Unit, Cambridge Biomedical Campus Cambridge United Kingdom

## Abstract

**Objective:**

Defective mitochondrial function attributed to optic atrophy 1 (*OPA1*) mutations causes primarily optic atrophy and, less commonly, neurodegenerative syndromes. The pathomechanism by which *OPA1* mutations trigger diffuse loss of neurons in some, but not all, patients is unknown. Here, we used a tractable induced pluripotent stem cell (iPSC)‐based model to capture the biology of OPA1 haploinsufficiency in cases presenting with classic eye disease versus syndromic parkinsonism.

**Methods:**

iPSCs were generated from 2 patients with OPA1 haploinsufficiency and 2 controls and differentiated into dopaminergic neurons. Metabolic profile was determined by extracellular flux analysis, respiratory complex levels using immunoblotting, and complex I activity by a colorimetric assay. Mitochondria were examined by transmission electron microscopy. Mitochondrial DNA copy number and deletions were assayed using long‐range PCR. Mitochondrial membrane potential was measured by tetramethylrhodamine methyl ester uptake, and mitochondrial fragmentation was assessed by confocal microscopy. Exome sequencing was used to screen for pathogenic variants.

**Results:**

OPA1 haploinsufficient iPSCs differentiated into dopaminergic neurons and exhibited marked reduction in OPA1 protein levels. Loss of OPA1 caused a late defect in oxidative phosphorylation, reduced complex I levels, and activity without a significant change in the ultrastructure of mitochondria. Loss of neurons in culture recapitulated dopaminergic degeneration in syndromic disease and correlated with mitochondrial fragmentation.

**Interpretation:**

OPA1 levels maintain oxidative phosphorylation in iPSC‐derived neurons, at least in part, by regulating the stability of complex I. Severity of OPA1 disease associates primarily with the extent of OPA1‐mediated fusion, suggesting that activation of this mechanism or identification of its genetic modifiers may have therapeutic or prognostic value. Ann Neurol 2018;83:915–925

Mitochondria form a dynamic network that responds to physiological signals and stressors by altering morphology and connectivity to adapt to metabolic demands in health and disease. A defect in mitochondrial function has been causatively linked to a variety of neurodegenerative diseases, the commonest being Parkinson's disease. Optic atrophy 1 (OPA1) is a dynamin‐like GTPase within the inner mitochondrial membrane, which maintains fusion^1^ and by assembling into oligomers, keeps the cristae junctions in a closed state.^2^ These core functions of OPA1 appear to be critical for adaptation to bioenergetic demands,^3^ the control of apoptosis by limiting cytochrome *c* mobilization from the cristae interior,^2^, ^4^ and, possibly, mitochondrial DNA (mtDNA) stability.^5^, ^6^ Despite these essential functions in model systems, OPA1 mutations in humans cause primarily a selective degeneration of retinal ganglion cells and optic atrophy, but 20% of patients develop a diffuse neurological syndrome.^6^, ^7^ Most human *OPA1* mutations are nonsense and frameshift mutations that encode truncated transcripts prone to degradation by nonsense‐mediated mRNA decay and could therefore produce haploinsufficiency. The extent of mutant *OPA1* transcript depletion in patients is variable, ranging from no change to an apparent ∼60% loss.^8^

Recently, missense mutations in *OPA1* causing decreased protein expression were associated with parkinsonism and cognitive decline,^9^ suggesting that under certain conditions, loss of OPA1 function may compromise dopaminergic cell viability. The mechanism by which loss of OPA1 leads to cellular dysfunction in human neurons and whether any of these are relevant to disease severity is unknown. Here, we investigated how loss of OPA1 attributed to haploinsufficiency impacts on induced pluripotent stem cell (iPSC)‐derived dopaminergic neurons from patients with syndromic parkinsonism and pure optic atrophy and asked in this tractable model whether specific functions of OPA1 may associate with syndromic disease.

## Materials and Methods

### Reprogramming Donor Fibroblasts to iPSCs

Participants were recruited to this study having given signed informed consent, which included mutation screening and derivation of human iPSC lines from skin biopsies (Ethics Committee: National Health Service, Health Research Authority, NRES Committee South Central, Berkshire, UK, who specifically approved this part of the study [REC 10/H0505/71]). The control iPSC lines, AH017‐7 and OX3‐9, were previously published.^11^, ^12^ iPSCs were derived from fibroblasts as described previously,^11^ using the SeVdp(KOSM)302L Sendai virus system.^13^ iPSC lines tested negative for mycoplasma using MycoAlert (Lonza, Basel, Switzerland).

### Assessment of Genome Integrity and Tracking

Genome integrity was assessed by Illumina Human CytoSNP‐12v2.1 beadchip array (300,000 markers), analyzed using KaryoStudio and GenomeStudio software (Illumina, San Diego, CA). Single‐nucleotide polymorphism (SNP) profiles in the iPSC lines were compared to the corresponding fibroblasts to confirm their origin and identity.

### PluriTest

RNA was extracted from iPSCs using an RNeasy kit (Qiagen, Hilden, Germany) for Illumina HT12v4 transcriptome array analysis. Image data files were uploaded to http://www.pluritest.org and scored for pluripotency as previously described.^14^

### Flow Cytometry

iPSC lines were assessed for expression of pluripotency markers by flow cytometry. Cells were lifted with TrypLE (Life Technologies, Carlsbad, CA), fixed with 4% paraformaldehyde (PFA), permeabilized in methanol at −20 °C, washed and stained in flow cytometry buffer with antibody, measured using fluorescent‐activated cell sorting (FACS) Calibur (Becton Dickinson, Franklin Lakes, NJ), and analyzed using FlowJo software (TreeStar, Inc., Ashland, OR). Antibodies used were TRA‐1‐60 (B119983, IgM‐488; BioLegend, San Diego, CA) and NANOG (D73G4, IgG‐647; Cell Signaling Technology, Beverly, MA)

### Exome Sequencing and Analysis

DNA was extracted from freshly collected blood samples and sequenced on Illumina HiSeq 2000. Reads (100 base pair [bp], paired‐end) were mapped to the reference genome (HG19) using BWA, and variants were called using the Genome Anaysis Toolkit (GATK) best practice guidelines. Variants were annotated using SnpEFF and filtered using SnpSift and GATK. We searched for genes that showed nonsynonymous coding variants, with a minor allele frequency (MAF) < 1% in public databases (<1% in the 1000 Genomes Project [1KG] and in the Exome Aggregation Consortium [ExAC] that were present only in the index case, but not his mother. We further filtered the candidate genes on the basis of the predicted impact of each variant by combined annotation‐dependent depletion score (>10) and the number of damaging mutations found in healthy individuals for each gene by gene‐damaging index. Candidate modifiers were annotated with their expression level in brain using the Gtex database and their haploinsifficiency score (http://nar.oxfordjournals.org/content/43/15/e101). Genes with known mitochondrial function were annotated using the MitoCarta database.

### Accession Numbers

SNP data sets and Illumina HT12v4 expression array data sets for the iPSC lines have been deposited in GEO, under Accession Number GSE94433.

### Generation of iPSC‐Derived Midbrain Dopaminergic Neurons

Dopaminergic neurons were derived using a previously published protocol with some modifications.^15^ iPSCs were seeded onto Geltrex coated six‐well plates, expanded until >80% confluency, and mTeSR1 media was changed to day 0 (D0) media (2 μM of A83‐01, 100nM of LDN in neural induction base medium). D1‐4 media contained 2 μM of A83‐01, 100nM of LDN, 300ng/ml of SHH C25II, 2 μM of purmorphamine, and 200ng/ml of FGF8a in neural induction base medium. CHIR‐99021 (3 μM) was added from day 3 until day 12. D5‐6 media contained 100nM of LDN, 300ng/ml of SHH C25II, 2 μM of purmorphamine, 3 μM of CHIR‐99021, and 200ng/ml of FGF8a in neural induction base medium. D7‐10 media contained 100nM of LDN and 3 μM of CHIR‐99021 in neural induction base medium. D11‐19 medium contained 20ng/ml of brain‐derived neurotrophic factor, 20ng/ml of glial cell line–derived neurotrophic factor, 1ng/ml of transforming growth factor beta 3, 10 μM of DAPT, 200 μM of ascorbic acid, and 500 μM of dibutyryl‐cAMP in neural differentiation media, and 1 μg/ml of of laminin was added from 17 to 25 days of differentiation. At D20, cells were replated into geltrex coated plates, or coverslips. From D21 media, changes were done with D11 to D19 components until analysis time points (D25, D45, and D65) were reached.

### Immunofluorescence Staining

iPSC‐derived neurons were fixed in 4% PFA, permeabilized in 0.3% TritonX‐100 in 2% bovine serum albumin, 3% goat serum containing phosphate‐buffered saline, and incubated with primary antibodies and fluorescently labeled secondary antibodies. The following primary antibodies were used: anti‐Tom20 (FL145, rabbit polyclonal; Santa Cruz Biotechnology, Santa Cruz, CA), anti‐TH (mouse monoclonal; Millipore, Billerica, MA), anti‐βIII tubulin/TUJ1 (mouse monoclonal, BioLegend; rabbit polyclonal from Abcam [Cambridge, MA]), anti‐FOXA2 (A12 clone, Santa Cruz Biotech). Images were obtained using UltraVIEW VoX Spinning Disk Confocal Microscope (PerkinElmer, Waltham, MA) and analyzed using CellProfiler. For dopaminergic makers, 30 images (63 × magnification) were analyzed for each marker per differentiation per line. For fragmentation and mitotracker measurements, 20 to 30 images (at 63×) were obtained per time point per clone per differentiation and >900 cells were analyzed per subject. For cell numbers, at least 20 images (20 × magnification) were obtained per time point per differentiation using immunofluorescence microscopy (696 × 520). Larger clusters that could not be quantified were excluded by the software, and, in total, >30,000 cells were counted per subject for cell death measurements.

### Electrophysiology

Voltage‐clamp recordings were obtained from iPSC‐derived neurons using an Axopatch 200B amplifier (Molecular Devices, San Jose, CA). For the current clamp recordings 130mM of KCl, 1 mM of MgCl_2_, 5mM of MgATP, 10mM of HEPES, and 0.5mM of EGTA (pH 7.3) solution was used. Recordings were obtained at room temperature, with a sampling rate of 5KHz, using the pClamp 10 acquisition software (Molecular Devices). Data were analysed using MatLab R2015A (The MathWorks, Inc., Natick, MA).

### mtDNA Copy Number and Deletion Levels Determination and Long‐Range PCR

mtDNA was amplified in two fragments of approximately 9.9 kilobases (kb) and 15.4 kb in length using Takara PrimeSTAR GXL DNA polymerase (Takara Bio Inc., Kusatsu, Japan). Primer sequences were as follows: 1F: CCCTCTCTCCTACTCCTG (bp 6,222–6,239), 1R: CAGGTGGTCAAG TATTTATGG (bp 16,133–16,153) and 2F: TTAAAACTCAAAGGACCTGGC (bp 1,157–1,177), 2R AGGGTGATAGACCTGTGATC (bp 19–1). qPCR was used to amplify mitochondrial genes MTND1 and MTND4 and the nuclear gene, B2M. Control samples with or without mtDNA deletions were included in all assays. All samples were assayed in triplicates per qPCR run. mtDNA copy number (CN) was calculated based on the relative abundance of MTND1 gene compared to the nuclear gene, B2M. mtDNA deletion levels were calculated using the comparative threshold cycle (Ct) method of MTND4 and MTND1 genes.^16^

### Metabolic Studies

Oxygen consumption rates (OCRs) were measured using the Seahorse XFe96 analyzer (Seahorse Bioscience, North Billerica, MA) in cells seeded on XFe96 microplates. The Mito stress test kit (Seahorse Bioscience) was used to monitor OCR. Three baseline recordings were made, followed by sequential injection of the ATP synthase inhibitor, oligomycin (3 μM), the mitochondrial uncoupler, carbonyl cyanide‐4‐(trifluoromethoxy)phenyl‐hydrazone (1.25 μM), and the respiratory chain inhibitors, antimycin A (0.5 μM) and rotenone (0.5 μM). Results were corrected for cell numbers using CyQuant (ThermoFisher Scientific, Waltham, MA). For complex I enzyme activity, lysates were incubated with a specific complex I antibody precoated on microplates (ab109721; Abcam) and activity was subsequently determined by following the oxidation of NADH to NAD^+^ using a colorimetric assay.

### Electron Microscopy

Cells on coverslips were fixed with prewarmed fixative (2.5% glutaraldehyde and 2% PFA in 0.1M of PIPES buffer; pH 7.2). Cells were incubated in 1% osmium tetroxide, overnight in 0.5% uranyl acetate (aqueous), and taken through a graded ethanol series and gradually infiltrated with Agar100 epoxy resin. Blocks were polymerized and submerged in liquid nitrogen. Ultrathin (90‐nm) sections were obtained using a Leica UC7 ultramicrotome with a diamond knife (DiATOME, Hatfield, PA) and placed on 200 mesh copper grids, then poststained with Reynold's lead citrate and imaged on a FEI Tecnai 12 transmission electron microscope (TEM) operated at 120kV using a Gatan OneView CMOS camera. Between 15 and 30 images per clone per differentiation were scored using ImageJ (NIH, Bethesda, MD) software.

### Statistical Analysis

The statistical analysis was performed using Prism (GraphPad Software Inc., La Jolla, CA). Data were analyzed using one‐way analysis of variance (ANOVA). Biological replicates (*n*) are defined as differentiations performed at least one cell‐split apart, which is generally 1 week. The number of replicates included two clones per subject each differentiated three times.

## Results

### Clinical and Genetic Information

**Figure 1:**
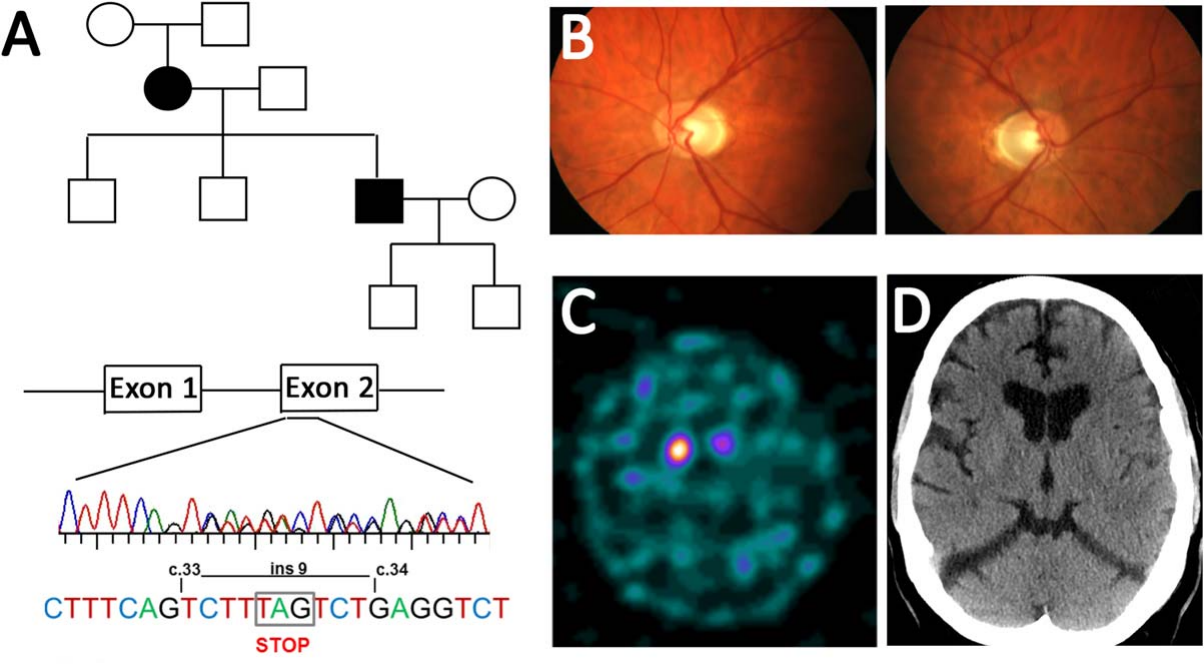
Clinical information. (A) Family tree of the index case who presented at the age of 46 with syndromic parkinsonism and his mother who manifested with pure optic atrophy until the age of 84. The OPA1 mutation consisted of a 9 base pair insertion introducing a stop codon in exon 2 leading to haploinsufficiency. (B) Bilateral optic atrophy in index case. (C) DaTScan showing evidence of asymmetric dopaminergic cell loss in the index case. (D) Recent CT brain of the index case showing mild generalised involutional changes without signal change within the basal ganglia. CT = computed tomography; OPA1 = optic atrophy 1. [Color figure can be viewed at http://www.annalsofneurology.org]

The proband is a 62‐year‐old male who presented at the age of 46 with 1‐year history of micrographia and unilateral tremor on a background of dominantly inherited optic atrophy since his mid‐thirties. On examination at presentation in 2001, he had a mild resting tremor, moderate rigidity, and bradykinesia on the right upper limb without any corticospinal, cerebellar features or autonomic involvement. He had a positive DaTScan showing asymmetric left‐sided dopaminergic loss (Fig 1). He did not respond to dopamine agonists or levodopa therapy and progressed significantly within 6 months with inability to write and unsteadiness attributed to a rigid right leg, requiring support with a stick. His examination revealed progression of the extrapyramidal signs with bilateral asymmetric involvement of both upper and lower limbs, worse on the right. Within 2 years of his initial diagnosis, he developed frequent falls, walked with a broad‐based festinant gait, and had impaired postural reflexes. There was no objective improvement with subcutaneous apomorphine. He became wheelchair bound within 6 years from the initial presentation. He stopped working at the age of 48. His estimated premorbid IQ was 110 and his cognitive profile, recognition memory, and non‐verbal memory as documented at 10 years postdiagnosis were in line with estimates of his premorbid ability. He developed some swallowing difficulties, occasional visual hallucinations, and suffered from low mood when assessed 14 years postdiagnosis. His brain magnetic resonance imaging (MRI) at presentation was normal. He had normal motor and sensory nerve conduction on neurophysiology. His mother suffered with progressive visual loss since the second decade of her life with documented optic atrophy and passed away at the age of 87. When assessed at the age of 84, she did not have any other neurological deficits and was very independent in her daily activities living alone despite her visual problems. During the last 3 years of her life, her condition deteriorated with balance problems and memory decline. She was using the wheelchair only during the last 12 months of her life. Genetic testing in both cases revealed a novel insertion in exon 2 of 3 in‐frame codons (c.33‐34ins9), the second of which is a stop codon predicted to result in *OPA1* haploinsufficiency (Fig 1A). Common *POLG* and mtDNA mutations were negative in blood, and no mutations were identified in genes implicated in familial parkinsonism. The second *OPA1* allele was sequenced in both cases and was normal without any of the intronic *OPA1* mutations that have been reported to act as modifiers in some cases with syndromic OPA1 disease.^10^ To identify candidate modifier genes, we performed whole‐exome sequencing searching for genes that showed nonsynonymous coding variants, with a MAF that were present only in the index case but not his mother. We identified a total of 54 variants in 54 genes that met these criteria. Nine of the genes identified (*ATG4C, CENPJ, ERCC3, SH3GLB2, TSC1, HNRNPU, PIN1, NT5M*, and *HSPBP1*) are expressed in neuronal tissues, but none of the variants identified are known to be pathogenic.

### Generation and Characterization of Human iPSC Lines

**Figure 2:**
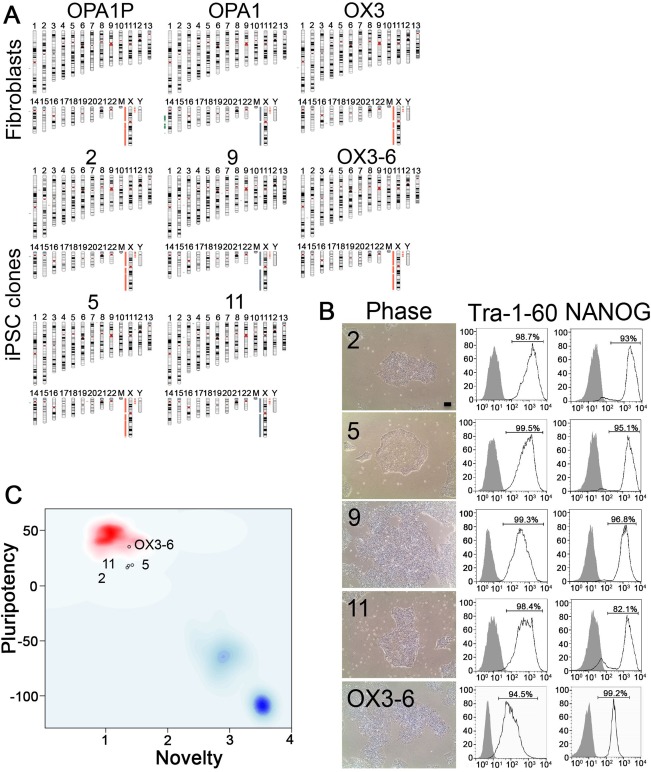
Generation of iPSC lines. (A) Karyograms for each iPSC line and original fibroblasts. (B) Human iPSC lines exhibited pluripotent stem cell‐like morphology by phase microscopy in feeder‐free culture; scale bar = 100 µm; Right panel, FACS analysis of iPSC for pluripotency markers Tra‐1‐60 and Nanog (black line); grey filled plot, isotype control. (C) PluriTest analysis showed that the iPSC lines cluster with pluripotent stem cells (top left quadrant). Each circle represents one iPSC line *y*‐axis pluripotency score, *x*‐axis novelty score. FACS = fluorescence‐activated cell sorting; iPSC = induced pluripotent stem cell. [Color figure can be viewed at http://www.annalsofneurology.org]

We generated two human iPSC clonal lines from fibroblasts obtained from each of the 2 patients with OPA1 haploinsufficiency and compared them to iPSC from 2 healthy controls (Table). Detailed characterization of the OPA1 lines and the newly derived healthy lines used in this study is shown in Figure 2. Genome integrity and tracking to the original patient sample was confirmed by Illumina SNP array (Fig 2A). All iPSC lines displayed embryonic stem cell–like morphology and expressed the pluripotency‐associated proteins, TRA‐1‐60 and Nanog (Fig 2B). Conformity to a pluripotent expression profile was shown by PluriTest (Fig 2C).

### Progressive Neuronal Loss in syndromic OPA1 Disease Is Recapitulated in iPSC‐Derived Cultures

**Figure 3:**
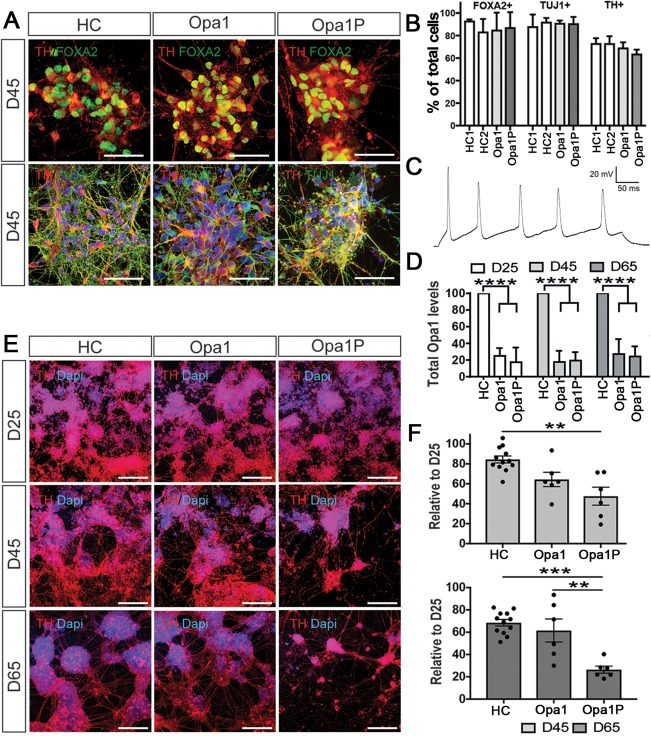
iPSC‐derived dopaminergic neurons from syndromic OPA1 parkinsonism recapitulate disease severity. (A) Immunostaining for TH, FOXA2, and TUJ1 was readily detected across controls and patient lines, scale bar 30 μm. (B) Quantification at D45 revealed equal percentage of FOXA2‐, TUJ1‐, and TH‐positive cells across lines. (C) Representative example of iPSC derived dopaminergic neuron at DIV65, showing repetitive spiking in response to a 400ms depolarizing pulse (D) Quantification of immunoblotted OPA1 protein revealed marked reduction in both patient lines at all time‐points tested. (E) Accelerated cell death was detected in clones of iPSC‐derived dopaminergic neurons from the patient with syndromic disease when cultured for D45 and D65, scale bar 150 μm. (F) Quantification of the percentage of cells remaining at D45 and D65 compared to D25. Data are mean ± SEM and biological replicates are defined as individual clone differentiations using two clones per subject each differentiated three times. HC = healthy control. Analysis was done using one‐way ANOVA (**p < 0.01; ***p < 0.001; ****p < 0.0001). ANOVA = analysis of variance; iPSC = induced pluripotent stem cell; OPA1 = optic atrophy 1; SEM = standard error of the mean.

To study the effect of the OPA1 haploinsufficinecy in the context of parkinsonism, two iPSC clones from each of the 2 controls and the 2 patients were each differentiated three times into dopaminergic neuronal cultures. Results from lines derived from the patient with syndromic OPA1 parkinsonism are denoted as Opa1P, whereas lines derived from the patient with optic atrophy are represented as Opa1. We confirmed by immunofluorescence that in all lines, dopaminergic neurons coexpressed the floor plate marker, FOXA2, with TH or β‐3 tubulin (TUJ1) with TH (Fig 3A). Differentiation efficiency, as assessed by the immunofluorescence for FOXA2, β‐3 tubulin, and TH, was similar across the genotypes used with approximately 80% of cells expressing β‐3 tubulin and 70% expressing TH (Fig 3B). At day in vitro (DIV) 65, current‐clamp recordings showed that neurons were able to fire repetitive action potentials in response to current injection (Fig 3C). Both patient lines exhibited markedly reduced OPA1 protein levels when compared to controls (Fig 3D). We found accelerated neuronal death in the Opa1P patient when compared to Opa1 or control lines at DIV 45 and 65 (Fig 3E,F), recapitulating the degeneration of neurons detected by DaTScan in the patient. The absence of a secondary pathogenic mutation on exome sequencing of patient DNA to explain this phenotypic difference between Opa1P and Opa1 lines suggests that syndromic OPA1 parkinsonism may arise from a modifier effect of the primary mitochondrial defect. We therefore asked how functions that were previously ascribed to OPA1 are manifested in Opa1 and Opa1P lines.

### OPA1 Haploinsufficiency Leads to Defective Oxidative Phosphorylation and Reduced Complex I Function in iPSC‐Derived Neurons

**Figure 4:**
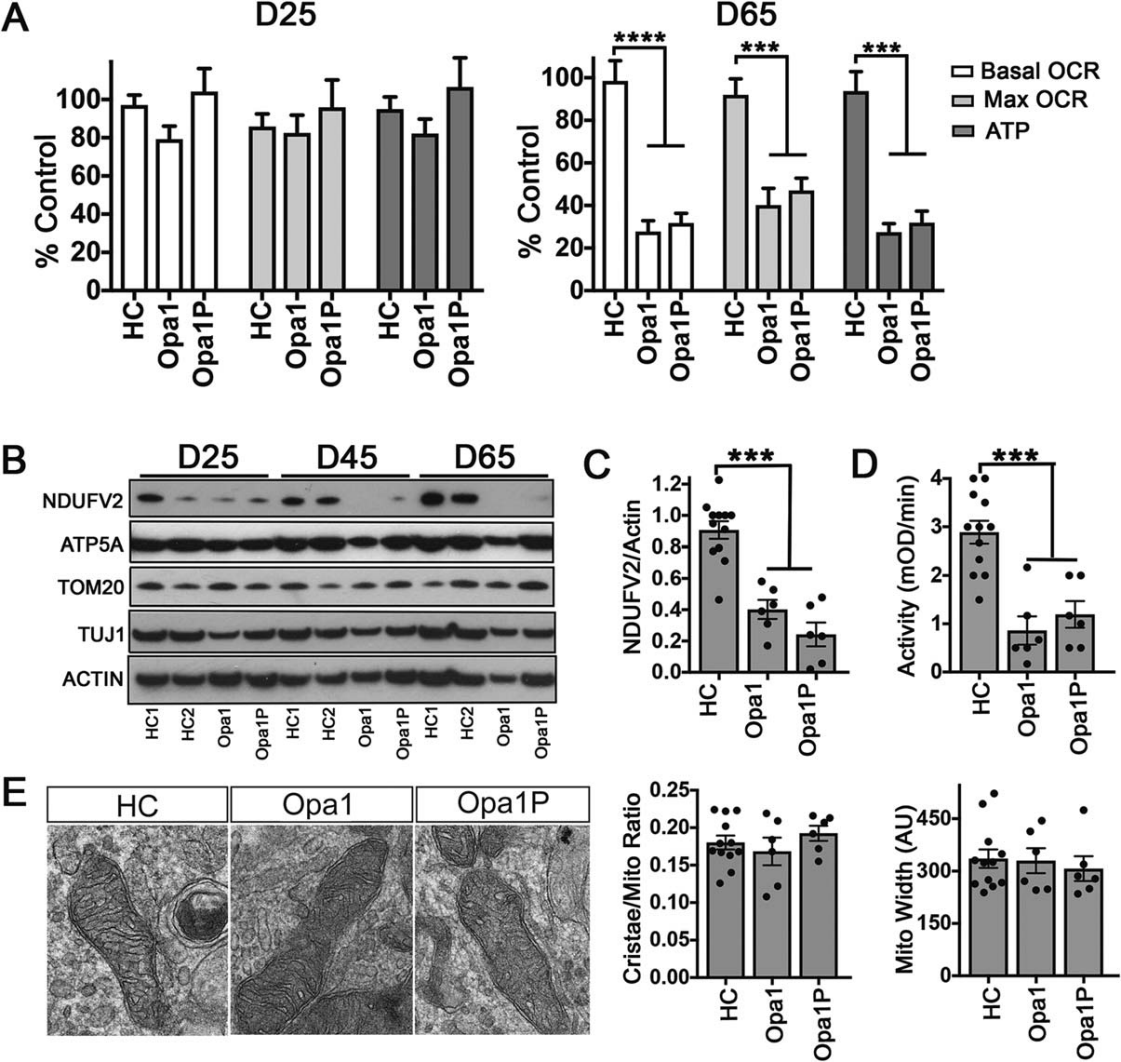
OPA1 haploinsufficiency causes progressive OXPHOS defect in iPSC‐derived neurons. (A) Extracellular flux analysis at D25 and D65 revealed a progressive decline in OCR, maximal respiration and ATP production in both patient lines. (B) Immunoblotting for mitochondrial proteins revealed a progressive decline in complex I in OPA1 haploinsufficient lines, which was quantified at D65 as shown in panel (C). (D) Complex I activity was reduced in OPA1 hapoinsufficient lines compared to controls at D65. (E) Morphometric analysis of the ratio of surface area of cristae to corresponding mitochondrial segment or mitochondrial width at D65. Each dot represents the average of at least 15 randomly selected images from each clone. Quantification in all panels is based on two clones per subject each differentiated three times. Data are mean ± SEM and analysed using One‐way ANOVA (*p < 0.05; **p < 0.01; ****p < 0.0001). ANOVA = analysis of variance; HC = healthy control; iPSC = induced pluripotent stem cell; OCR = oxygen consumption rate; OPA1 = optic atrophy 1; SEM = standard error of the mean.

**Figure 5:**
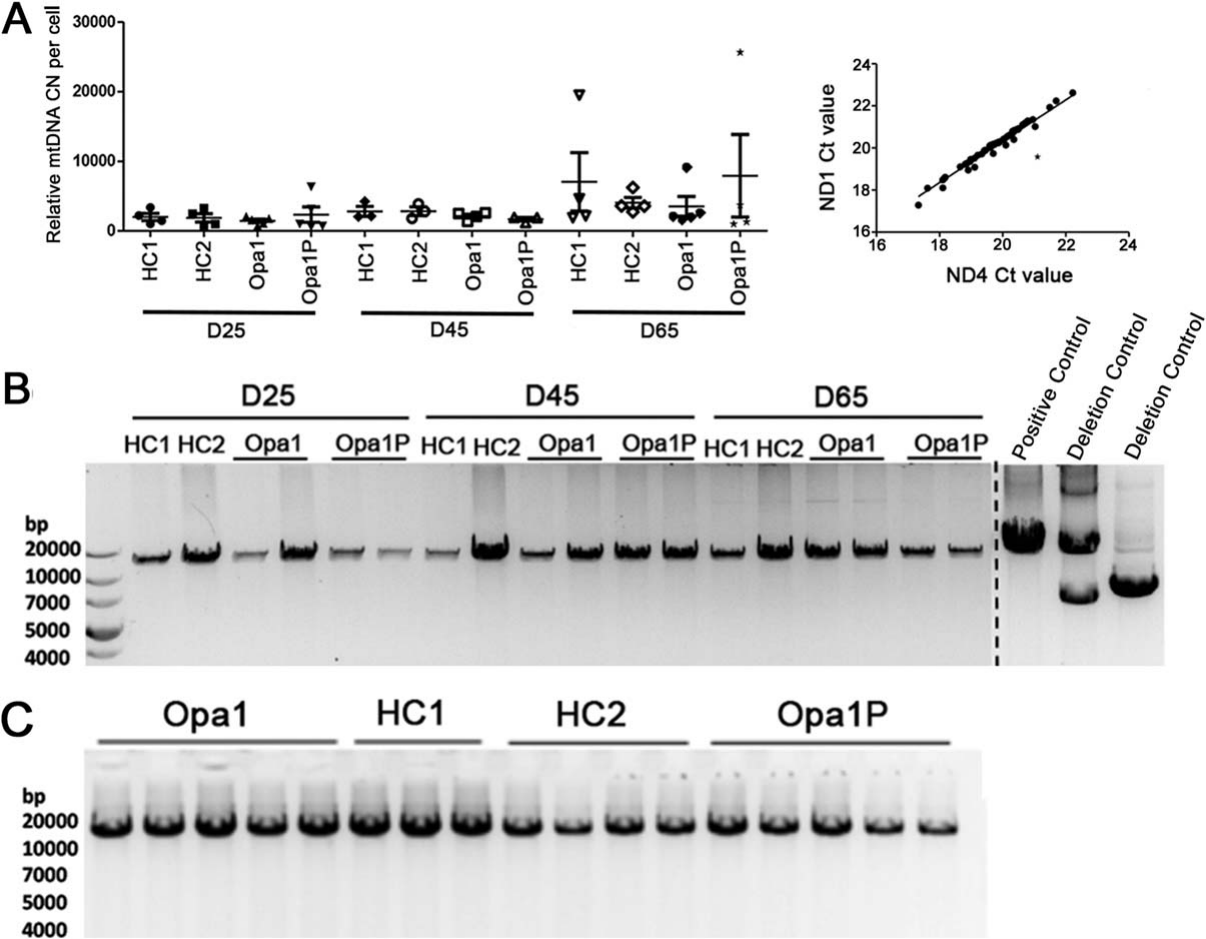
iPSC and iPSC‐derived neurons do not exhibit mtDNA deletions or copy number variations. (A) Left panel: MtDNA copy number (CN) per cell measurement showing the mean with standard error of the mean calculated from six independent assays using two clones per subject. Right panel: Correlation of ND1 and ND4 Ct values in neurons showing a strong correlation (Pearson r = 0.9887) and implying that no deletions in mtDNA were present. An asterisk represents a deletion control. Gels show representative images of long‐range PCR analysis on DNA isolated from iPSC‐derived neurons (B) and iPSC lines (C). bp = base pair; Ct = threshold cycle; iPSC = induced pluripotent stem cell; mtDNA = mitochondrial DNA; Opa1 = optic atrophy 1.

To assess whether OPA1 haploinsufficiency impairs bioenergetic demand, we performed extracellular flux analysis of OCR. This revealed that loss of OPA1 in iPSC‐derived neurons led to lower basal and maximal OCR compared to controls at a late (DIV65), but not early (DIV25), time point, mechanistically suggesting a progressive defect in electron transport in both Opa1P and Opa1 lines (Fig 4A). Bioenergetic failure was associated with loss of complex I as assessed by NDUFV2 levels relative to actin (Fig 4B,C) and a defect in complex I activity (Fig 4D) in both patients compared to controls. Notably, mitochondrial mass based on Tom20 levels relative to actin and complex V based on ATP5A levels relative to actin were unchanged across time points and lines (Fig 4B). Despite this clear functional deficit, we did not detect overt changes in the morphology of the mitochondria or cristae when assessed by TEM at either early or late time points (DIV25/45/85, not shown) and illustrated here at DIV65 (Fig 4E). mtDNA analysis did not reveal any deletions in the patient‐derived neurons (Fig 5) as previously reported in skeletal muscle.^5^, ^6^ Thus, reduced OPA1 protein levels in iPSC‐derived neurons cause a delayed defect in oxidative phosphorylation, which is most likely at the level of complex I.

### Mitochondrial Fragmentation in iPSC‐Derived Neurons Correlates With Clinical Severity in Patients

**Figure 6:**
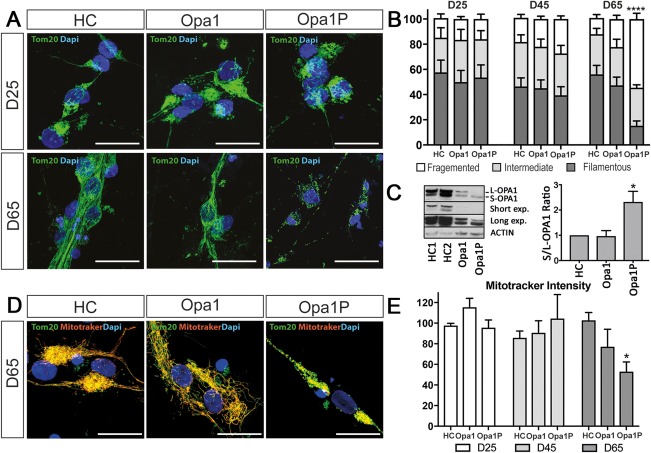
Mitochondrial fragmentation in iPSC‐derived neurons associates with severity of clinical phenotype in patients. (A) Progressive fragmentation of Tom20‐positive mitochondria was detected by D65 in Opa1P lines. (B) Quantification of the percentage of neurons with fragmented mitochondria networks. (C) The abundance of cleaved (short) S‐OPA1 isoform was increased in Opa1P neurons. (D–E) Mitochondria in Opa1P exhibited reduced uptake of mitotracker. Quantification in all panels is based on two clones per subject each differentiated three times. Data are mean ± SEM and analysed using one‐way ANOVA (*p < 0.05; **p < 0.01; ****p < 0.0001). Scale bar, 20 μm. ANOVA = analysis of variance; HC = healthy control; iPSC = induced pluripotent stem cell; Opa1 = optic atrophy 1; SEM = standard error of the mean.

To investigate the relevance of OPA1‐mediated mitochondrial fusion in our model, we assessed the mitochondrial network by quantifying the percentage of neurons with fragmented or filamentous Tom20‐positive mitochondria. This analysis showed that by DIV65, fragmentation was increased in the Opa1P lines compared to either Opa1 or controls (Fig 6A,B). Accordingly, the abundance of the cleaved (short) S‐OPA1 isoform, which typically accumulates in cells with fragmented mitochondria, was increased in Opa1P neurons (Fig 6C). When assessed as individual networks using the ratio of mitotracker Red to Tom20, the fragmented mitochondrial segments in Opa1P neurons exhibited reduced uptake and thus impaired function at DIV65 (Fig 6D,E).

## Discussion

Despite the multifactorial role of OPA1 in mitochondrial health, loss of its function commonly causes autosomal‐dominant optic atrophy and, only under certain conditions, leads to a systemic mitochondrial disease. What is its critical function in human neurons and how this is linked to neuronal vulnerability to cell death is currently unclear. Using an iPSC‐based model, we showed that loss of OPA1 causes a late‐onset deficit in oxidative phosphorylation and a differential loss of mitochondrial fusion, which associates with disease severity. Unlike earlier OPA1 knockdown studies in cell lines,^3^, ^17^ we did not observe marked disorganization of the morphology of the mitochondria or their cristae in patient‐derived neurons. This discrepancy is most likely attributed to the fact that previous studies in cell lines have examined the consequences of acute and/or complete depletion of OPA1 within 24 to 72 hours^3^, ^17^ instead of pathophysiologically relevant OPA1 levels as in our patient‐derived neuronal model, where the defect developed over 45 to 65 days. This scenario is in agreement with the slowly progressive focal neurological phenotype in most patients with OPA1 haploinsufficiency.^7^ It is possible that under conditions of neuronal stress, the ultrastructural morphology of OPA1 haploinsufficient mitochondria is disorganized before cell death and our TEM studies have not captured such events. Instead, our data suggest that a late defect in complex I may account for the reduction of electron transport across the respiratory chain as evidenced by the decreased maximal respiration in mutant neurons and the reduction of complex I levels and activity. Functional changes in complex I were previously reported in fibroblasts with OPA1 haploinsufficiency^18^ or the p.G488R mutation^9^ and in models of acute OPA1 depletion, where respiratory efficiency was impaired when mitochondria were energized specifically with the complex I substrates, glutamate/malate.^17^ Therefore, in human iPSC‐derived neurons, OPA1 levels are important for the maintenance of oxidative phosphorylation, at least partly, by regulating the stability of complex I.

In the context of syndromic disease, impaired regulation of oxidative phosphorylation and complex I deficiency was not sufficient to explain the differential viability of dopaminergic neurons between Opa1P and Opa1 lines. Interestingly, clinicopathological studies in patients with *POLG* mutations also found that complex I deficiency did not correlate with parkinsonian features.^19^ Given that fragmentation has been associated with apoptosis,^20^ the cell death in culture could be, at least partly, determined by the exacerbated mitochondrial fragmentation in Opa1P lines, which also exhibited increased proteolytic processing of L‐OPA1 to the short isoform. Proteolytic cleavage of L‐OPA1 is regulated by either YME1L1, which is under metabolic control^21^ or OMA1, which is activated upon various stress insults, resulting in the complete degradation of L‐OPA1 and mitochondrial fragmentation.^22^, ^23^ Interestingly, in a mouse model of neurodegeneration, L‐OPA1 promoted neuronal survival without affecting cristae shape, whereas stress‐induced OMA1 activation and L‐OPA1 cleavage increased mitochondrial fragmentation and promoted neuronal death.^24^

Further investigation is required to determine which cellular stressors or adaptive mechanisms promote or oppose OPA1‐mediated mitochondrial fragmentation in syndromic OPA1 disease and potentially other common diseases. Our initial whole‐exome analysis did not identify a secondary mutation of known pathogenicity, but does not exclude the contribution of multiple minor genetic determinants such as effects on autophagy, which is activated in cells carrying a mutant *OPA1* allele^9^, ^25^ or microtubule‐associated transport, which is important for axonal mitochondrial motility. This withstanding, our finding that key cellular phenotypes are reproduced in vitro suggests, for the first time, that syndromic disease may arise from genetic modifiers that exacerbate the mitochondrial defect, which, at least in dopaminergic neurons, manifested as OPA1‐mediated mitochondrial fragmentation. It is possible that different mechanisms operate in other subtypes of syndromic OPA1‐mediated disease. Nevertheless, understanding how L‐OPA1 processing varies in syndromic OPA1 and common diseases of dopaminergic cell loss such as Parkinson's and promoting L‐OPA1 stability (eg, by OMA1 inhibition) could pave the way for novel targeted therapies against mitochondrial dysfunction in neurodegeneration. More broadly, our study demonstrates how deep‐phenotyping of a rare disease using iPSCs can help contextualize key functions of essential mitochondrial proteins in the study of disease severity, which is an important step toward personalized neurology, and suggests that mapping of genetic modifiers could have prognostic significance.

## Author Contributions

G.K.T. contributed to the conception and design of the study and drafting of the manuscript. M.J., M.M., A.M., T.Z., S.W., C.E.Z., M.K.‐A., C.B. D.S., P.F.C., S.C., and G.K.T contributed to data acquisition and analysis and drafting of figures.

## Potential Conflicts of Interest

Nothing to report.

**Table 1:** Summary of the Lines Used in This Study

ID	Sex	Age at Biopsy	No. of iPSC clones	Original ID	Characterization
Control 1	Male	49	2	OX3‐6 OX3‐9	This study Dafinca et al 2016
Control 2	Female	67	2	AH017‐3 AH017‐7	Handel et al 2016
OPA1	Female	84	2	OPA1‐9 OPA1‐11	This study
OPA1P	Male	62	2	OPA1P‐2 OPA1P‐5	This study

Two clones per subject were used each differentiated three times.

iPSC = induced pluripotent stem cell; OPA1 = optic atrophy 1.
